# Effect of tumor necrosis factor inhibitors on risk of cardiovascular disease in patients with axial spondyloarthritis

**DOI:** 10.1186/s13075-022-02836-4

**Published:** 2022-06-13

**Authors:** Oh Chan Kwon, Min-Chan Park

**Affiliations:** Division of Rheumatology, Department of Internal Medicine, Yonsei University College of Medicine, Eonjuro, Gangnam-gu, Seoul, 06273 South Korea

**Keywords:** Axial spondyloarthritis, Cardiovascular risk, Tumor necrosis factor inhibitor, Inflammation

## Abstract

**Background:**

Axial spondyloarthritis (axSpA) is associated with an increased risk of cardiovascular disease. We aimed to evaluate the effect of tumor necrosis factor inhibitors (TNFis) on the risk of cardiovascular disease in patients with axSpA.

**Methods:**

This retrospective study included 450 patients with axSpA without pre-existing cardiovascular disease. The outcome was incident cardiovascular disease (myocardial infarction or stroke) after the diagnosis of axSpA. The effect of TNFis on cardiovascular risk was analyzed in the total study population and in an inverse probability of treatment weighting (IPTW)-adjusted population. Cox proportional hazards models were used to estimate the hazard ratios (HRs) and 95% confidence intervals (95% CIs) for cardiovascular disease, according to exposure to TNFis.

**Results:**

Of the 450 patients, 233 (51.8%) and 217 (48.2%) patients were and were not exposed to TNFis, respectively. Twenty cardiovascular diseases occurred during 2868 person-years of follow-up (incidence rate: 6.97/1000 person-years). In the total study population, exposure to TNFis was associated with a reduced cardiovascular risk when adjusted for traditional cardiovascular risk factors (HR 0.30, 95% CI 0.10–0.85, *p* = 0.024). However, when time-averaged erythrocyte sedimentation rate and C-reactive protein were additionally adjusted, this association was attenuated and lost statistical significance (HR 0.37, 95% CI 0.12–1.12, *p* = 0.077). Furthermore, in the IPTW-adjusted population, exposure to TNFis showed no significant reduction in cardiovascular risk (HR 0.60, 95% CI 0.23–1.54, *p* = 0.287).

**Conclusions:**

Although controlling inflammation through TNFis could be beneficial in cardiovascular risk reduction, our data indicate no TNFi-specific reduction in cardiovascular risk in patients with axSpA.

## Background

Axial spondyloarthritis (axSpA) is a chronic inflammatory arthritis that mainly affects the axial skeleton [1, 2]. In addition to the traditional cardiovascular risk factors (age, sex, hypertension, dyslipidemia, smoking, and obesity), inflammation is associated with accelerated atherosclerosis and is an important contributor to cardiovascular risk [3–5]. The increased risk of cardiovascular disease in inflammatory joint diseases including ankylosing spondylitis (AS) has been well established [5]. A meta-analysis assessing the risk of myocardial infarction (MI) and stroke in patients with AS showed that compared with those without AS, patients with AS have a higher risk of MI (odds ratio [OR] 1.60, 95% confidence interval [CI] 1.32–1.93) and stroke (OR 1.50, 95% CI 1.39–1.62) [6]. Moreover, a population-based study showed that AS is associated with a higher risk of cardiovascular mortality (OR 1.36, 95% CI 1.13–1.65) [7]. A similar association was also reported in a meta-analysis assessing risk of comorbidities in patients with axSpA, which includes both AS and non-radiographic axSpA; the authors reported a higher risk of ischemic heart disease (OR 1.51, 95% CI 1.21–1.87) and stroke (OR 1.30, 95% CI 1.04–1.62) [8]. Given that patients with axSpA are at higher risk of cardiovascular morbidity and mortality, careful monitoring and treatment of modifiable cardiovascular risk factors are important in these patients.

Tumor necrosis factor (TNF)-α, a pro-inflammatory cytokine involved in the pathogenesis of axSpA [9], is also involved in the initiation and progression of atherosclerosis and in rupture of atherosclerotic plaque [10, 11]. TNF inhibitors (TNFis) are widely used in patients with axSpA and are effective in controlling inflammation [12–17]. Considering the role of TNF-α in inflammation and atherosclerosis, the use of TNFis may lead to a reduction in cardiovascular risk in patients with axSpA. Indeed, some studies showed that TNFis reduce subclinical atherosclerosis in patients with AS [18, 19]. However, these studies used subclinical atherosclerosis as a surrogate outcome, and whether the use of TNFis in patients with axSpA is associated with a reduction in cardiovascular disease as an outcome remains unclear. Here, we aimed to assess whether the exposure to TNFis is associated with a lower risk of cardiovascular disease in patients with axSpA.

## Methods

### Patients

In this retrospective cohort study, patients with axSpA who visited a tertiary referral hospital in Seoul, Korea between January 1, 2010, and December 31, 2011, were initially selected. All patients fulfilled the Assessment of SpondyloArthritis international Society classification criteria for axSpA [20]. Patients with a history of MI or stroke or chronic kidney disease (CKD) prior to the diagnosis of axSpA were excluded. Those who were followed up for less than a year were also excluded. The occurrence of incident cardiovascular disease in each patient was retrospectively reviewed. The index date for each patient was the date of the first visit between January 1, 2010, and December 31, 2011. Patients were followed up from the index date to the date of occurrence of cardiovascular disease, discontinuation of TNFis (for patients who were on TNFis at the index date), initiation of TNFis (for those who were not on TNFis at the index date), last visit, or June 30, 2021, whichever came first. This study was approved by the institutional review board (IRB) of Gangnam Severance Hospital (IRB No: 3-2021-0328). Owing to the retrospective nature of this study, the requirement for informed consent was waived.

### Exposure to TNFis

TNFis were used in patients who had an inadequate response despite treatment with non-steroidal anti-inflammatory drugs (NSAIDs; either non-selective NSAIDs or selective cyclooxygenase-2 [COX-2] inhibitors), with or without conventional synthetic disease-modifying anti-rheumatic drugs (csDMARDs), for at least 3 months. Patients who were on TNFis at the index date were classified as the “TNFi exposed” group. Those who were naïve to TNFis at the index date were classified as the “TNFi not exposed” group. None of the patients in both groups were receiving or had received other biologic DMARDs (interleukin-17A inhibitors) or targeted synthetic DMARDs (Janus kinase inhibitors). As per the definition of follow-up, patients in the TNFi exposed group were exposed to TNFis throughout the whole follow-up duration, while patients in the TNFi not exposed group were not exposed to TNFis throughout the follow-up duration.

### Covariates

The following covariates at the index date of axSpA were collected: age, sex, presence of hypertension, diabetes mellitus, and dyslipidemia, smoking status (current smoker or not), body mass index (BMI), symptom duration of axSpA, HLA-B27 positivity, fulfillment of the radiological criterion of the 1984 modified New York criteria [21], presence of syndesmophyte, prevalence of peripheral manifestations, psoriasis, uveitis, and inflammatory bowel diseases, erythrocyte sedimentation rate (ESR), and C-reactive protein (CRP) levels. Regarding medication, use of naproxen, non-selective NSAIDs other than naproxen, selective COX-2 inhibitors, csDMARDs (methotrexate and sulfasalazine), aspirin, angiotensin-converting enzyme inhibitor (ACEi) or angiotensin II receptor blocker (ARB), sodium-glucose cotransporter-2 inhibitors (SGLT2 inhibitors), and statin within 6 months prior to the index date and during follow-up was reviewed. A medication was considered “used” if the patient had been exposed to the medication for at least 3 months. Time-averaged ESR and CRP during follow-up were calculated as indices of inflammatory burden during the follow-up. Time-averaged ESR and CRP were defined as the mean values of ESR and CRP levels, respectively, determined every 3 months.

### Definition of cardiovascular disease

Cardiovascular disease was defined as an incident MI or stroke that occurred after the index date with a 6-month lag. To assess whether cardiovascular disease have occurred in each patient, we reviewed whether each patient had undergone coronary angiography, cardiac enzyme tests, or brain magnetic resonance imaging during the follow-up duration. MI events were defined as an MI that was diagnosed by cardiologists based on the coronary angiography and cardiac enzyme levels. Stroke events were defined as a stroke diagnosed by neurologists based on brain imaging studies.

### Statistical analysis

For the description of patient characteristics, continuous variables were expressed as mean (± standard deviation) or median (interquartile range) for parametric or non-parametric variables, respectively, and categorical variables were expressed as numbers (%). For comparison between two groups, independent two sample *t*-test or Mann-Whitney *U* test was used for continuous variables, and *χ*^2^ test or Fisher’s exact test for categorical variables. Cox proportional hazards regression analysis was performed to estimate the hazard ratios (HRs) and 95% CIs for cardiovascular disease according to the exposure to TNFis. The proportional hazards assumption was tested by examining log (−log [survival]) curves and Schoenfeld partial residuals: no relevant violations were found. Univariable Cox regression analysis was performed initially, followed by five multivariable Cox regression analyses. Multivariable model 1 was adjusted for traditional cardiovascular risk factors (age, sex, hypertension, diabetes mellitus, dyslipidemia, smoking, and BMI). Multivariable model 2 was adjusted for time-averaged ESR and CRP in addition to the traditional cardiovascular risk factors. Multivariable model 3 was adjusted for baseline ESR and CRP in addition to the traditional cardiovascular risk factors. Multivariable model 4 was adjusted for use of NSAIDs and csDMARDs. Multivariable model 5 was adjusted for use of aspirin, ACEi or ARB, SGLT2 inhibitor, and statin. To assess whether there is a different effect when monoclonal TNFis are analyzed as a separate group, we excluded patients who were exposed to etanercept in the TNFi exposed group and additionally conducted the multivariable models 1–5.

As patients were not randomly assigned to the two groups, inverse probability of treatment weighting (IPTW)-adjusted analysis was conducted to reduce potential confounding by indication. The propensity score used in the IPTW-adjusted analysis was estimated by multiple logistic regression analysis including all covariates in Table 1. In the IPTW-adjusted population, continuous variables were expressed as weighted mean (± standard error), and categorical variables were expressed as weighted numbers (%). An IPTW-adjusted Cox proportional hazards regression analysis was performed to estimate the HR and 95% CI for cardiovascular disease according to the exposure to TNFis. A *p*-value of < 0.05 was considered statistically significant. All analyses were conducted using SAS version 9.4 (SAS Institute Inc., Cary, NC, USA).

**Table 1:** Comparison of characteristics according to exposure to TNFis

	Total (*N* = 450)	TNFi exposed (*N* = 233)	TNFi not exposed (*N* = 217)	*P* value
Traditional cardiovascular risk factors				
Age, year, mean (± SD)	36.4 (±13.0)	36.6 (±13.1)	36.3 (±13.0)	0.811
Male, *n* (%)	343 (76.2)	179 (76.8)	164 (75.6)	0.756
Hypertension, *n* (%)	63 (14.0)	39 (16.7)	24 (11.1)	0.083
Diabetes mellitus, *n* (%)	35 (7.8)	22 (9.4)	13 (6.0)	0.172
Dyslipidemia, *n* (%)	74 (16.4)	42 (18.0)	32 (14.7)	0.348
Current Smoker, *n* (%)	62 (13.8)	39 (16.7)	23 (10.6)	0.059
BMI, kg/m^2^, mean (± SD)	23.8 (±3.6)	23.9 (±3.6)	23.7 (±3.7)	0.655
AxSpA-related factors				
Symptom duration, years, median (IQR)	5.0 (1.9–10.1)	5.3 (2.1–10.3)	4.3 (1.3–10.1)	0.079
HLA-B27 positive^a^, *n* (%)	356 (82.6)	187 (85.0)	169 (80.1)	0.179
Radiographic axSpA, *n* (%)	365 (81.1)	190 (81.5)	175 (80.6)	0.807
Syndesmophyte present, *n* (%)	123 (27.3)	60 (25.8)	63 (29.0)	0.435
Peripheral manifestations, *n* (%)	228 (50.7)	124 (53.2)	104 (47.9)	0.262
Psoriasis, *n* (%)	23 (5.1)	14 (6.0)	6 (4.1)	0.370
Uveitis, *n* (%)	81 (18.0)	44 (18.9)	37 (17.1)	0.613
Inflammatory bowel diseases, *n* (%)	10 (2.2)	7 (3.0)	3 (1.4)	0.341
ESR, mm/h, median (IQR)	17.5 (8.0–40.0)	17.0 (8.0–39.8)	18.5 (8.0–40.3)	0.978
CRP, mg/L, median (IQR)	4.1 (0.8–13.9)	5.1 (1.1–15.8)	3.3 (0.8–10.5)	0.038
Medications				
Non-selective NSAIDs^b^, *n* (%)	313 (69.6)	169 (72.5)	144 (66.4)	0.155
Naproxen, *n* (%)	194 (43.1)	98 (42.1)	96 (44.2)	0.641
Selective COX-2 inhibitors, *n* (%)	395 (87.8)	216 (92.7)	179 (82.5)	0.001
Methotrexate, *n* (%)	67 (14.9)	53 (22.7)	14 (6.5)	< 0.001
Sulfasalazine, *n* (%)	373 (82.9)	201 (86.3)	172 (79.3)	0.049
Aspirin, *n* (%)	11 (2.4)	5 (2.1)	6 (2.8)	0.671
ACEi or ARB, *n* (%)	46 (10.2)	26 (11.2)	20 (9.2)	0.497
SGLT2 inhibitors, *n* (%)	6 (1.3)	3 (1.3)	3 (1.4)	> 0.999
Statin, *n* (%)	60 (13.3)	35 (15.0)	25 (11.5)	0.275
Time-averaged ESR, mm/h, median (IQR)	11.3 (6.0–20.7)	10.3 (5.9–21.4)	12.7 (6.4–21.0)	0.329
Time-averaged CRP, mg/L, median (IQR)	2.2 (0.9–4.7)	2.1 (0.9–4.3)	2.4 (1.0–5.7)	0.168

*TNFi* tumor necrosis factor inhibitor, *BMI* body mass index, *HLA* human leukocyte antigen, *axSpA* axial spondyloarthritis, *ESR* erythrocyte sedimentation rate, *CRP* C-reactive protein, *ACEi* angiotensin-converting enzyme inhibitor, *ARB* angiotensin II receptor blocker, *SGLT2* sodium-glucose cotransporter-2, *NSAIDs* non-steroidal anti-inflammatory drugs, *COX-2* cyclooxygenase-2

^a^Patients (*n* = 19) with missing data excluded

^b^Non-selective NSAIDs other than naproxen

## Results

### Comparison of characteristics between the two groups

A total of 450 patients with axSpA were included in the analysis. The mean age at the index date was 36.4 (±13.0) years, and 343 (76.2%) patients were male. Of the 450 patients, 233 (51.8%) and 217 (48.2%) patients were classified as TNFi exposed and TNFi not exposed, respectively. Of the 233 patients in the TNFi exposed group, 142 (60.9%), 61 (26.2%), and 30 (12.9%) patients were exposed to adalimumab, etanercept, and infliximab, respectively. The comparison of characteristics between the two groups is shown in Table 1. There were no significant differences in traditional cardiovascular risk factors between the two groups. In terms of axSpA related factors, CRP at the index date was significantly higher in the TNFi exposed group than in the TNFi not exposed group (5.1 [1.1–15.8] mg/L vs. 3.3 [0.8–10.5] mg/L, *p* = 0.038). In terms of medication, a higher proportion of patients was exposed to non-selective NSAIDs (91.4% vs. 80.2%, *p* = 0.001), selective COX-2 inhibitors (92.7% vs. 82.5%, *p* = 0.001), methotrexate (22.7% vs. 6.5%, *p* < 0.001), and sulfasalazine (86.3% vs. 79.3%, *p* = 0.049) in the TNFi exposed group than the TNFi not exposed group. Time-averaged ESR and CRP did not differ between the two groups.

### Comparison of occurrence of cardiovascular disease between the two groups

Incident cardiovascular disease occurred in 20 patients (MI, 12 patients; stroke, 8 patients) during 2868 person-years of follow-up (incidence rate: 6.97/1000 person-years). One cardiovascular disease occurred during the 6-month lag and was not included for analysis. Cardiovascular disease occurred less commonly in the TNFi exposed group than in the TNFi not exposed group (2.6% vs. 6.5%, *p* = 0.046), despite the longer duration of follow-up (6.3 [3.4–10.2] years vs. 4.7 [2.8–8.2] years, *p* = 0.002) (Table 2). The duration of TNFi treatment in the “TNFi exposed” group before index date was 2.1 (1.1–3.8) years.

**Table 2:** Comparison of outcomes according to the exposure to TNFis

	Total (*N* = 450)	TNFi exposed (*N* = 233)	TNFi not exposed (*N* = 217)	*P* value
Incident cardiovascular disease, *n* (%)	20 (4.44)	6 (2.6)	14 (6.5)	0.046
Duration of follow-up, years, median (IQR)	5.5 (2.9–9.4)	6.3 (3.4–10.2)	4.7 (2.8–8.2)	0.002
Time to events, years, median (IQR)	3.9 (1.8–7.2)	3.3 (2.0–8.4)	4.4 (1.7–7.0)	> 0.999

*TNFi* tumor necrosis factor inhibitor, *ESR* erythrocyte sedimentation rate, *CRP* C-reactive protein

### Risk of cardiovascular disease according to the exposure to TNFis

The estimated HR for cardiovascular disease according to the exposure to TNFis is reported in Table 3. In the univariable analysis, the TNFi exposed group had an HR of 0.35 (95% CI 0.13–0.93, *p* = 0.034) for cardiovascular disease compared with the TNFi not exposed group. After adjusting for traditional cardiovascular risk factors, the TNFi exposed group was still significantly associated with a lower risk of cardiovascular disease (HR 0.30, 95% CI 0.10–0.85, *p* = 0.024) compared with the TNFi not exposed group (multivariable model 1). However, with additional adjustment for time-averaged ESR and CRP, the effect size was attenuated and lost statistical significance (multivariable model 2: HR 0.37, 95% CI 0.12–1.12, *p* = 0.077). Similarly, the statistical significance was also lost in multivariable model 3, where baseline ESR and CRP were adjusted in addition to the traditional cardiovascular risk factors (HR 0.42, 95% CI 0.14–1.21, *p* = 0.107). On the other hand, in multivariable model 4 (HR 0.36, 95% CI 0.13–0.98, *p* = 0.045) and model 5 (HR 0.29, 95% CI 0.11–0.81, *p* = 0.017), where inflammatory burden was not adjusted, exposure to TNFis was significantly associated with a lower risk of cardiovascular disease. When patients who were exposed to etanercept were excluded, and only those who were exposed to monoclonal TNFis were analyzed as a separate group, similar results were observed (Table 4).

**Table 3:** Estimation of hazard ratios for cardiovascular disease according to exposure to TNFis

	HR (95% CI)	*P* value
Univariable analysis		
TNFi not exposed	1.00 (reference)	
TNFi exposed	0.35 (0.13–0.93)	0.034
Multivariable model 1 adjusted for traditional cardiovascular risk factors^a^		
TNFi not exposed	1.00 (reference)	
TNFi exposed	0.30 (0.10–0.85)	0.024
Multivariable model 2 adjusted for traditional cardiovascular risk factors^a^ and time-averaged ESR and CRP		
TNFi not exposed	1.00 (reference)	
TNFi exposed	0.37 (0.12–1.12)	0.077
Multivariable model 3 adjusted for traditional cardiovascular risk factors^a^ and baseline ESR and CRP		
TNFi not exposed	1.00 (reference)	
TNFi exposed	0.42 (0.14–1.21)	0.107
Multivariable model 4 adjusted for use of NSAIDs and csDMARDs		
TNFi not exposed	1.00 (reference)	
TNFi exposed	0.36 (0.13–0.98)	0.045
Multivariable model 5 adjusted for use of aspirin, ACEi or ARB, SGLT2 inhibitor, and statin		
TNFi not exposed	1.00 (reference)	
TNFi exposed	0.29 (0.11–0.81)	0.017

*TNFi* tumor necrosis factor inhibitor, *HR* hazard ratio, *CI* confidence interval, *ESR* erythrocyte sedimentation rate, *CRP* C-reactive protein, *NSAIDs* non-steroidal anti-inflammatory drugs, *csDMARDs* conventional synthetic disease-modifying anti-rheumatic drugs, *ACEi* angiotensin-converting enzyme inhibitor, *ARB* angiotensin II receptor blocker, *SGLT2* sodium-glucose cotransporter-2

^a^Traditional cardiovascular risk factors: age, sex, hypertension, diabetes mellitus, dyslipidemia, smoking, and body mass index

**Table 4:** Estimation of hazard ratios for cardiovascular disease according to exposure to monoclonal TNFis

	HR (95% CI)	*P* value
Univariable analysis		
TNFi not exposed	1.00 (reference)	
TNFi exposed	0.37 (0.14–0.97)	0.043
Multivariable model 1 adjusted for traditional cardiovascular risk factors^a^		
TNFi not exposed	1.00 (reference)	
TNFi exposed	0.30 (0.11–0.87)	0.026
Multivariable model 2 adjusted for traditional cardiovascular risk factors^a^ and time-averaged ESR and CRP		
TNFi not exposed	1.00 (reference)	
TNFi exposed	0.38 (0.13–1.14)	0.084
Multivariable model 3 adjusted for traditional cardiovascular risk factors^a^ and baseline ESR and CRP		
TNFi not exposed	1.00 (reference)	
TNFi exposed	0.42 (0.15–1.22)	0.112
Multivariable model 4 adjusted for use of NSAIDs and csDMARDs		
TNFi not exposed	1.00 (reference)	
TNFi exposed	0.38 (0.14–0.99)	0.049
Multivariable model 5 adjusted for use of aspirin, ACEi or ARB, SGLT2 inhibitor, and statin		
TNFi not exposed	1.00 (reference)	
TNFi exposed	0.30 (0.11–0.83)	0.021

*TNFi* tumor necrosis factor inhibitor, *HR* hazard ratio, *CI* confidence interval, *ESR* erythrocyte sedimentation rate, *CRP* C-reactive protein, *NSAIDs* non-steroidal anti-inflammatory drugs, *csDMARDs* conventional synthetic disease-modifying anti-rheumatic drugs, *ACEi* angiotensin-converting enzyme inhibitor, *ARB* angiotensin II receptor blocker, *SGLT2* sodium-glucose cotransporter-2

^a^Traditional cardiovascular risk factors: age, sex, hypertension, diabetes mellitus, dyslipidemia, smoking, and body mass index

### IPTW-adjusted analysis

A comparison of the characteristics between the TNFi exposed group and the TNFi not exposed group in the IPTW-adjusted population is summarized in Table 5. There were no significant differences in the characteristics between the two groups. In contrast to the unweighted total study population, occurrence of cardiovascular disease did not differ between the two groups (3.42% and 6.25%, respectively, *p* = 0.403). In the IPTW-adjusted Cox proportional hazards regression analysis, no significant association was observed between TNFis exposure and risk of cardiovascular disease (Table 6: HR 0.60, 95% CI 0.23–1.54, *p* = 0.287).

**Table 5:** Comparison of characteristics according to exposure to TNFis in the IPTW-adjusted population

	TNFi exposed (*N* = 207.57)	TNFi not exposed (*N* = 192)	*P* value
Traditional cardiovascular risk factors			
Age, year, weighted mean (SE)	36.083 (1.587)	36.188 (0.956)	0.955
Male, weighted *n* (%)	148.90 (71.73)	142.00 (73.96)	0.749
Hypertension, weighted *n* (%)	25.52 (12.30)	23.00 (11.98)	0.934
Diabetes mellitus, weighted *n* (%)	13.59 (6.54)	12.00 (6.25)	0.924
Dyslipidemia, weighted *n* (%)	32.44 (15.63)	28.00 (14.58)	0.821
Current Smoker, weighted *n* (%)	20.91 (10.07)	20.00 (10.42)	0.924
BMI, kg/m^2^, weighted mean (SE)	23.815 (0.351)	23.676 (0.268)	0.752
AxSpA-related factors			
Symptom duration, year, weighted mean (SE)	6.926 (0.591)	7.045 (0.573)	0.885
HLA-B27 positive, weighted *n* (%)	177.37 (85.45)	152.00 (79.17)	0.162
Radiographic axSpA, weighted *n* (%)	168.50 (81.18)	152.00 (79.17)	0.695
Syndesmophyte present, weighted *n* (%)	66.18 (31.88)	52.00 (27.08)	0.493
ESR, mm/h, weighted mean (SE)	26.038 (2.423)	27.156 (1.837)	0.713
CRP, mg/L, weighted mean (SE)	8.866 (1.165)	10.555 (1.700)	0.413
Medications			
Non-selective NSAIDs, weighted *n* (%)	144.55 (69.64)	155.00 (80.73)	0.105
Selective COX-2 inhibitors, weighted *n* (%)	166.84 (80.38)	159.00 (82.81)	0.742
Methotrexate, weighted *n* (%)	16.21 (7.81)	13.00 (6.77)	0.737
Sulfasalazine, weighted *n* (%)	152.30 (73.37)	154.00 (80.21)	0.336
Aspirin, weighted *n* (%)	7.29 (3.51)	5.00 (2.60)	0.704
ACEi or ARB, weighted *n* (%)	20.71 (9.98)	19.00 (9.90)	0.982
SGLT2 inhibitors, weighted *n* (%)	2.12 (1.02)	3.00 (1.56)	0.624
Statin, weighted *n* (%)	25.91 (12.48)	22.00 (11.46)	0.808
Time-averaged ESR, mm/h, weighted mean (SE)	16.354 (1.769)	15.149 (0.850)	0.540
Time-averaged CRP, mg/l, weighted mean (SE)	4.901 (1.103)	4.617 (0.442)	0.811
Incident cardiovascular disease, weighted *n* (%)	7.11 (3.42)	12.00 (6.25)	0.403

*TNFi* tumor necrosis factor inhibitor, *BMI* body mass index, *HLA* human leukocyte antigen, *axSpA* axial spondyloarthritis, *ESR* erythrocyte sedimentation rate, *CRP* C-reactive protein, *ACEi* angiotensin-converting enzyme inhibitor, *ARB* angiotensin II receptor blocker, *SGLT2* sodium-glucose cotransporter-2, *NSAIDs* non-steroidal anti-inflammatory drugs, *COX-2* cyclooxygenase-2

**Table 6:** Risk for cardiovascular disease according to exposure to TNFis in the IPTW-adjusted population

	HR (95% CI)	*P*-value
TNFi not exposed	1.00 (reference)	
TNFi exposed	0.60 (0.23–1.54)	0.287

*TNFi* tumor necrosis factor inhibitor, *IPTW* inverse probability of treatment weighting, *HR* hazard ratio, *CI* confidence interval

## Discussion

In this study, we evaluated the influence of TNFis on the risk of cardiovascular disease in patients with axSpA, and showed that exposure to TNFis is not specifically associated with a reduction in the risk of cardiovascular disease.

The effect of TNFis on the risk of cardiovascular disease has been extensively studied in patients with rheumatoid arthritis (RA) [22, 23]. A meta-analysis of 28 studies showed that the use of TNFis is associated with a reduced risk of MI (relative risk 0.59, 95% CI 0.36–0.97) and stroke (relative risk 0.57, 95% CI 0.35–0.92) [23]. However, the effect of TNFis on the risk of cardiovascular disease in patients with axSpA has been less robustly studied. There is some evidence showing that TNFis may have differential effects on arterial stiffness in patients with AS and RA [24–27]. Use of TNFis reduced arterial stiffness in patients with RA [24, 25], but not in patients with AS [26, 27]. Given this discrepancy in the effect of TNFis on arterial stiffness between AS and RA, the beneficial effect of TNFis on risk of cardiovascular disease in patients with RA may not be directly applicable to patients with axSpA, as shown in our study.

A meta-analysis of 17 studies assessing the risk of MI and stroke in patients with AS reported a higher risk of MI and stroke in patients with AS than controls [6]. In that meta-analysis, the incidence rate of MI or stroke in patients with AS was 6.00/1000 person-years [6], which is comparable to the incidence rate in our study population (6.97/1000 person-years), thus supporting the external validity of our study population.

In contrast to our present finding, a recent study reported that the use of TNFis is associated with a reduced risk of cardiovascular disease in patients with SpA [28]. However, in that study, patients with SpA were heterogeneous, including substantial proportion (19.4%) of patients with psoriatic arthritis [28]. Moreover, the exposure to TNFis was not defined as use throughout the whole follow-up, but as use at any period during the follow-up [28], which leaves the possibility of misclassification of exposure. Therefore, by the previous study [28], it is still unclear whether the exposure to TNFis is associated with a reduced risk of cardiovascular disease in patients with axSpA. In our study, only patients with axSpA were included, and patients in the TNFi exposed group were exposed to TNFis throughout the follow-up duration, which excludes the possibility of misclassification of exposure. Based on our finding, use of TNFis itself does not seem to reduce the risk of cardiovascular disease in patients with axSpA.

Considering that TNFis were initiated only in patients whose inflammation was not well controlled with the use of NSAIDs with or without csDMARDs, patients in the TNFi exposed group inherently have a higher inflammatory burden than those in the TNFi not exposed group. Given that inflammation is one of the main contributors of cardiovascular risk [5], we can assume that the TNFi exposed group had a higher risk of cardiovascular disease prior to the initiation of TNFis than the TNFi not exposed group. Hence, an alternative interpretation of our results is that the use of TNFis in the TNFi exposed group resulted in a reduction in cardiovascular risk to a level similar to that of the TNFi not exposed group. Importantly, the time-averaged ESR and CRP (i.e., inflammatory burden) throughout the follow-up were similar between patients who were and were not exposed to TNFis, indicating that inflammation was effectively controlled with the use of TNFis in the TNFi exposed group. Therefore, although our data (multivariable model 2 and IPTW-adjusted analysis) showed no significant TNFi-specific reduction in cardiovascular risk, by effectively suppressing the inflammation, TNFis could still have a beneficial effect on cardiovascular risk. Notably, the exposure to TNFis was significantly associated with a reduced risk of cardiovascular disease in multivariable model 1, in which traditional cardiovascular risk factors were adjusted, but not the inflammatory burden throughout the follow-up. However, after additional adjustment for the inflammatory burden throughout the follow-up (multivariable model 2), this association was weakened and lost statistical significance. These results indicate that controlling inflammation, rather than TNFi use per se, is more important in reducing the risk of cardiovascular disease in patients with axSpA.

Confounding by indication is an important issue that needs to be considered when comparing patients exposed and not exposed to a particular drug. In our study population, we excluded patients with underlying CKD to minimize confounding by indication, as the presence of CKD is an important factor that affects axSpA treatment. For example, NSAIDs, the first-line medication for the treatment of axSpA [29, 30], cannot be used in these patients, whereas TNFis are relatively safe and well tolerated in patients with CKD [31]. Hence, inclusion of patients with CKD could be a major source of confounding by indication. Moreover, to further reduce confounding by indication, we performed IPTW-adjusted analysis, where characteristics were all well balanced between the two groups. The finding of multivariable model 2 was also observed in the IPTW-adjusted analysis. Therefore, we presume that our finding is not likely the result of confounding by indication.

There are some limitations to be noted in our study. First, this was a retrospective observational study. Although multiple potential confounders were adjusted using multivariable analysis and IPTW-adjusted analysis, confounding by unmeasured covariates cannot be fully excluded. For instance, data on physical activity, which could affect the risk of cardiovascular disease [5], were not available. We also lack data on family history of cardiovascular disease and data on previous smoking or dosage (pack years). Second, disease activity parameters including Bath Ankylosing Spondylitis Disease Activity Index, Ankylosing Spondylitis Disease Activity Score, and magnetic resonance of sacroiliac joints were not available in a number of patients. Third, there is a possibility that some events might have been missed if the event was treated in another hospital. However, as the incidence rate of MI or stroke in our study was comparable to a previous meta-analysis [6], we presume that the number of events missed would be low. Fourth, the number of events was relatively small, and we were unable to analyze MI and stroke separately. Further prospective controlled studies are needed to confirm our finding.

## Conclusions

In conclusion, exposure to TNFis was not associated with a reduction in risk of cardiovascular disease in patients with axSpA. Although a beneficial effect on cardiovascular risk through the reduction of inflammation could be expected with the use of TNFis, there was no TNFi-specific reduction in risk of cardiovascular disease in patients with axSpA.

## Data Availability

All data generated or analyzed during this study are included in this article.

## Abbreviations

AxSpA: Axial spondyloarthritis
AS: Ankylosing spondylitis
MI: Myocardial infarction
OR: Odds ratio
CI: Confidence interval
TNF: Tumor necrosis factor
TNFi: Tumor necrosis factor inhibitor
CKD: Chronic kidney disease
NSAIDs: Non-steroidal anti-inflammatory drugs
COX-2: Cyclooxygenase-2
csDMARDs: Conventional synthetic disease-modifying anti-rheumatic drugs
BMI: Body mass index
ESR: Erythrocyte sedimentation rate
CRP: C-reactive protein
ACEi: Angiotensin-converting enzyme inhibitor
ARB: Angiotensin II receptor blocker
SGLT2: Sodium-glucose cotransporter-2
HR: Hazard ratio
IPTW: Inverse probability of treatment weighting
RA: Rheumatoid arthritis
